# A novel case of prolonged Ifosfamide encephalopathy and long-term treatment with methylene blue: a case report and review of literature

**DOI:** 10.1186/s12887-022-03144-1

**Published:** 2022-02-02

**Authors:** Gabriel Chain, Mudit Kalia, Karen Kestenbaum, Lara Pappas, Anna Sechser-Perl, Gadi Abebe Campino, Nibal Zaghloul

**Affiliations:** 1 Department of Pediatrics, The Children’s Hospital at Saint Peter’s University Hospital, 254 Easton Avenue, New Brunswick, NJ 08901 USA; 2 Division of Pediatric Hematology/Oncology, The Children’s Hospital at Saint Peter’s University Hospital, New Brunswick, NJ 08901 USA; 3 Pediatric Hemato-Oncology division, Safra Children’s Hospital, Sheba Medical Center, Ramat Gan, Israel

**Keywords:** Somnolence, Ifosfamide, Methylene blue, Encephalopathy

## Abstract

**Background:**

Encephalopathy following Ifosfamide treatment is a well-described phenomenon that is typically treated with Methylene Blue (MB). Chloroacetaldehyde, a potentially neurotoxic metabolite of Ifosfamide is hypothesized to cause this encephalopathy. Current guidelines for treatment is to stop Ifosfamide and provide supportive care. MB acts to inhibit Chloroacetaldehyde formation and has been described as a therapy and prophylaxis for Ifosfamide-encephalopathy. MB is effective within 30 min and lasts up to 3 days. Prolonged encephalopathy and MB therapy has not been described in the literature as lasting longer than 30 days following treatment.

**Case presentation:**

We present the case of an 11-year-old female with autistic spectrum disorder and recurrent episodes of severe somnolence for 7 months following Ifosfamide therapy for her Non-Germinomatous Germ Cell Tumor (GCT). Periods of somnolence occurred prior to receiving cranial RT. Administration of MB gave immediate but limited response, with resolution of somnolence lasting 1-2 days between administrations. The somnolence could not be explained by neuroimaging or laboratory evaluation, but EEG indicated persistent encephalopathy.

**Conclusion:**

A literature review determines that neurotoxicity is a side effect of Ifosfamide, but this effect has not been described persisting longer than 30 days. Our case continued to require treatment with MB for 7 months following cessation of therapy. We report these novel clinical findings, and hypothesize that there could be a genetic/metabolic component linking this reaction to Ifosfamide with the case patient’s pre-existing autism. This possible association may also correlate to the already-established link between autism and the development of GCTs. This hypothesis leads to further discussion on the suitable usage of Ifosfamide in children with co-morbidities and the necessity of screening prior to its usage.

## Background

Germ Cell Tumors (GCT) account for 3% pediatric brain tumors and are subcategorized into Germinomas (G) and Non-Germinomatous (NG) GCT [1]. Most NGGCT involve the pineal or pituitary glands [2]. Pineal tumors often present with symptoms of hydrocephalus including lethargy, vomiting and mental status changes and pituitary tumors often present with polyuria or growth disruption.

Ifosfamide is a chemotherapeutic agent, which is a synthetic analog of cyclophosphamide. Side effects of Ifosfamide can include hair loss, bladder irritation and central nervous system (CNS) toxicity [3, 4]. These CNS toxicities include encephalopathy, lethargy and personality changes. During the metabolism of Ifosfamide, Chloroacetaldehyde is produced which is a potentially neurotoxic metabolite that crosses the blood-brain barrier and has been suggested as the etiology behind Ifosfamide encephalopathy [5].

The incidence of CNS toxicity following Ifosfamide therapy is estimated between 10 to 20 % [2]. High doses (>2G/M2/Day) of Ifosfamide correlate to higher rates of adverse effects, however, encephalopathy can be demonstrated at any dose and specific blood levels of Ifosfamide have not been clearly linked to the development of neurotoxicity. CNS toxicity usually presents acutely following drug administration and subsequently resolves following drug discontinuation [5]. Certain studies have demonstrated the effects lasting for up to 30 days following treatment [6]. Current guidelines for Ifosfamide encephalopathy include treatment cessation, supportive care and the use of Methylene Blue (MB) as a therapeutic and prophylactic treatment. Risk factors for Ifosfamide-encephalopathy include hypoalbuminemia and previous CNS disease [7].

MB is a therapy for Ifosfamide-encephalopathy and research suggests MB’s mechanism involves inhibition of monoamine oxidase which is involved in Chloroacetaldehyde formation. Potential adverse effects of MB include discoloration of the skin, vomiting and hemolytic anemia [8]. MB is effective within 30 min and can last up to 3 days.

## Case presentation

We present the case of an 11 yr female with a background of autism spectrum disorder of moderate severity (which presented in early childhood) who presented with 4 months of cognitive changes. Brain MRI detected a pineal tumor with hydrocephalus and spinal metastasis. GCT markers beta-HCG and alpha-fetoprotein (AFP) were elevated within the Cerebrospinal fluid (CSF). They underwent right frontal VP (ventriculoperitoneal) shunt insertion and tumor pathology reported an NGGCT. She was initiated onto the high-risk treatment arm: COG ACNSO122, which includes 6 cycles of induction chemotherapy (alternating cycles of Carboplatin/Etoposide and Ifosfamide/Etoposide and craniospinal radiation with boost to the tumor bed. Each cycle of Ifosfamide included 1800 mg/m^2^/day for 5 days. The patient’s baseline physical exam was unremarkable.

During the 4th course of chemotherapy (Ifosfamide/Etoposide) the patient developed sequelae of Ifosfamide encephalopathy which included drowsiness, confusion and somnolence. As per protocol for Ifosfamide toxicity (grade 2/3), treatment was started with MB (IV, 1 mg/kg, BID). Within hours of initiation, patient orientation returned to baseline. Multidisciplinary discussion was held to discuss the risks/benefit of continued Ifosfamide treatment and a decision was made to continue treatment with close observation and continued use of MB. During the 6th cycle (Ifosfamide/Etoposide, without concurrent use of the anti-emetic Aprepitant), 4 months after treatment initiation, MB was prophylactically used from Day − 1. On day 4 of this cycle, however, the patient developed somnolence. CT and MRI brain were normal, EEG demonstrated mild encephalopathy. Due to concerns of early hydrocephalus, VP shunt revision was performed, however, the somnolence persisted only resolving following MB treatment.

Ifosfamide metabolites, ammonia levels, thiamine levels, lumbar puncture and blood gas were all unchanged from their baseline. AFP/HCG, following initial elevation at diagnosis, then normalized and remained so on serial analysis, EEG continued to demonstrate mild encephalopathy both prior to, and following MB. Levetiracetam was trialed with no benefit noted.

During the radiation course, 7 months following diagnosis, the patient required multiple readmissions/reviews for recurrent episodes of somnolence. Shunt revision did not alter these episodes. CT and MRI did not note new pathology and serum AFP and B-HCG values continued to be within normal limits. EEG continued to demonstrate encephalopathy. During all these episodes, MB was used and within 30 min of initiation, the patient was at baseline. Physical exam demonstrated generalized reduced awakening with no other focal neurological findings.

After discussion, radiation therapy was initiated under close observation and no clinical deterioration was reported. Following this course of treatment, the patient was fully alert and orientated. They underwent further neurocognitive testing and they continue to have episodes of recurrent somnolence, however, there were significant cognitive deficits affecting behavior and memory. MRI of brain and spine showed no evidence of disease and blood/CSF markers remained negative. An “Invitae” genetic predisposition work-up was completed (including oncologic panel for germ-cell tumor predisposition and metabolic panels which included assessments for inborn errors of metabolism), which thus far does not note any significant findings. The patient continues to be in remission without evidence of disease.

## Discussion and conclusion

Ifosfamide-related encephalopathy is a phenomenon well described in the literature. The first reported case of Ifosfamide encephalopathy and MB treatment was documented in 1996 [9] and describes IE as occurring between 48 h and 30 days following treatment [10]. Prolonged Ifosfamide encephalopathy and prolonged treatment are novel findings that have not been described in the pediatric population [5]. A case series does describe two reports of prolonged Ifosfamide encephalopathy in an adult population, including a 41 year old woman who developed cognitive dysfunction following Ifosfamide infusion [11]. She was not treated with MB and her neurologic abnormalities persisted beyond 10 years. Encephalopathy can occur at any doses of Ifosfamide, however higher doses do predispose to a higher incidence of adverse effects.

Due to our case patient’s diagnosis of autism, we reviewed the literature for any correlation between the diagnosis of autism and the presence or severity of Ifosfamide encephalopathy. Whilst no specific data is published describing this correlation, there is established data associating Autism Spectrum Disorder (ASD) with the development of GCTs [12]. Therefore, we hypothesize that a diagnosis of ASD may also predispose to the toxic effects of Ifosfamide. Similarly, whilst no single physiological pathway is validated for this link, we propose that several plausible theories exist, for example the established effect of Chloroacetaldehyde on Glutathione, imbalance of which is a suggested a pathway to ASD [13, 14]. This hypothesis may indicate a need for more extensive preliminary screening of comorbid candidates requiring Ifosfamide. We acknowledge that this hypothesis is currently only supported by sparse observational reports, but we hope that this case will prompt others to report similar findings.

We also consider that neurocognitive disorders (such as autism spectrum disorder), may be confounders when analyzing the presentation of ‘somnolence’. We note, for example, that autism is a risk factor for sleep disturbance [15] and this can lead to neuropsychiatric complications. Our case patient and caregiver did not report significant sleep disturbance and overnight EEG was not indicative of sleep pathology. Polysomnography may help to qualify these episodes of ‘somnolence’.

The neuroprotective effects of MB was evaluated in a case review of 52 patients, which concluded that MB is an effective treatment for Ifosfamide encephalopathy [16]. The study also demonstrated that prophylactic treatment with MB leads to fewer and milder cases of Ifosfamide encephalopathy [17]. The mechanism of Ifosfamide encephalopathy centers around a metabolite, Chloroacetaldehyde, which is hypothesized to be inhibited via treatment with MB. Chloroacetaldehyde is typically eliminated within a 24-h period [18]. To investigate if delayed elimination of Chloroacetaldehyde is causing the observed symptoms, a metabolome would need to be performed on the patient. If metabolic signatures of Chloroacetaldehyde are identified following cessation of Ifosfamide, this would support the hypothesis that prolonged Ifosfamide encephalopathy is caused by increased retention of Chloroacetaldehyde. Blood levels of Ifosfamide in our patient were noted to be normal, however specific levels have not been validated when linked to encephalopathy.

In conclusion, Ifosfamide-induced encephalopathy is a well-described toxicity which usually lasts for a period of days to weeks following treatment. MB is described as a therapy for this encephalopathy, but prolonged encephalopathy and treatment with MB after several months of cessation of treatment with Ifosfamide has not been previously reported. We highlight this case in order contribute to the ongoing research on the usage of Ifosfamide in children with co-morbidities and the potential value of genetic or metabolic screening prior to its use.

## Data Availability

The datasets used and/or analyzed during the current study are available from the corresponding author on reasonable request.

## Abbreviations

MB: Methylene Blue
GCT: Germ Cell Tumor
G: Germinomatous
NG: Non-Germinomatous
CNS: Central Nervous System
CSF: Cerebrospinal Fluid
VP: Ventriculoperitoneal
ASD: Autism Spectrum Disorder
EEG: Electroencephalogram
